# Multimodal Imaging Approach to MEN-1 Syndrome-Associated Tumors

**DOI:** 10.3390/diagnostics15091164

**Published:** 2025-05-03

**Authors:** Alice Carli, Elisa Boffa, Matteo Bonatti, Marco Chincarini, Maria Vittoria Davì, Giulia A. Zamboni

**Affiliations:** 1 Institute of Radiology, Department of Diagnostics and Public Health, Policlinico GB Rossi, University of Verona, 37134 Verona, Italy; 2 Department of Radiology, Hospital of Bolzano (SABES-ASDAA), Teaching Hospital of Paracelsus Medical University (PMU), 39100 Bolzano, Italy; matteo.bonatti@sabes.it; 3 Endocrinology, Emergency Medicine, Policlinico GB Rossi, AOUI Verona, 37134 Verona, Italy; davi.mariavittoria@gmail.com

**Keywords:** MEN-1, pancreas, parathyroid, pituitary gland, CT, MRI, functional imaging

## Abstract

Multiple endocrine neoplasia type 1 (MEN-1) is an autosomal dominant inherited syndrome characterized by a genetic predisposition for the development of specific hormone-secreting tumors. Effective diagnosis and management of MEN-1 require genetic testing, regular surveillance, and imaging follow-up to detect and monitor tumor growth or recurrence and to plan for surgical intervention. The aim of this narrative review is to provide an overview of the current imaging modalities and their role in the diagnosis and follow-up of patients affected by MEN-1, focusing on the detection and characterization of associated neoplasms. The knowledge of the most frequent MEN-1 associated neoplasms and their imaging features is crucial for an accurate diagnosis, management, and treatment.

## 1. Introduction

Multiple endocrine neoplasia (MEN) syndromes are a group of genetic familiar diseases characterized by hyperplasia and the malignant transformation of several endocrine glands. There are four known MEN syndromes (MEN-1, MEN-2a, MEN-2b, and MEN-4) based on the underlying genetic defects, and each type is characterized by a different combination of endocrine gland tumors [1]. Multiple endocrine neoplasia 1 (MEN-1) is the most prevalent form. Effective diagnosis and management of MEN-1 syndrome require genetic testing, regular surveillance, imaging procedures to detect and monitor tumor growth or recurrence, and, potentially, surgical intervention. The aim of this narrative review is to provide an overview of the current imaging modalities and their role in the diagnosis and follow-up of MEN-1 patients, focusing on the detection and characterization of associated neoplasms.

## 2. MEN-1

MEN1 was first described in 1953 by Paul Wermer, from whom the alternative name of “Wermer syndrome” derives [2].

The disease has an estimated prevalence of 2–3 per 100,000; there is no sex predilection, and the age of onset varies from early childhood to advanced age, with a peak incidence between 20 and 40 years [3].

It is an autosomal dominant condition with high penetrance caused by a genetic defect of a tumor suppressor gene located on chromosome 11 that encodes the nuclear protein menin, which regulates cell growth and division. Hence, its mutation increases the risk of tumor development [4].

More than 500 known mutations can affect the gene; depending on the specific mutation, there may be a higher risk of more aggressive neoplasms. Additionally, there are reported cases of sporadic MEN-1 syndrome where no identifiable gene mutation is found [3,5].

A negative genetic result does not necessarily exclude MEN-1 syndrome in a patient since tests do not cover all the possible mutations of the involved gene. If the suspicion of MEN-1 disease is strong despite negative genetic tests, it is still recommended to follow the screening program, like patients with a genetically diagnosed disease, and asymptomatic first-degree relatives should also be evaluated.

**Figure 1:**
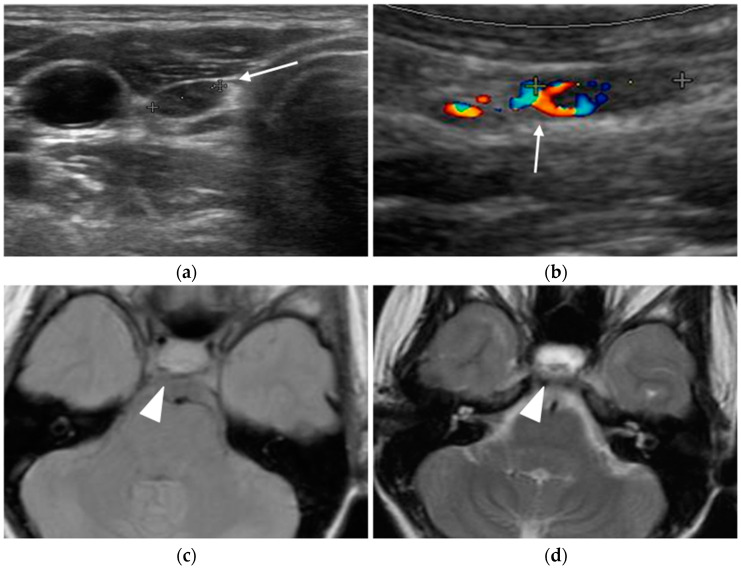

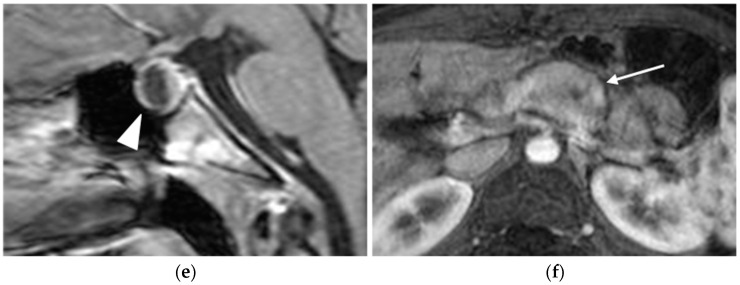
Young female MEN-1 patient, aged 19 at initial diagnosis. Ultrasound revealed a parathyroid adenoma (arrow in **a**,**b**) and MRI highlighted a pituitary macroadenoma (arrowhead in **c**–**e**) and a pancreatic NET (arrow in **f**).

The most common neoplasms associated with MEN-1 syndrome are parathyroid tumors, pancreaticoduodenal neuroendocrine tumors (NETs), and anterior pituitary tumors (Figure 1).

In addition, affected patients may develop adrenal cortical tumors, carcinoid tumors (bronchial, thymic, and gastric), and nonendocrine skin tumors, such as facial angiofibromas, lipomatous tumors, collagenomas, and dermatofibromas. The development of multiple facial angiofibromas, which occurs in 85–90% of affected patients, is a highly suggestive feature of MEN-1 syndrome [6,7].

The leading causes of death in MEN-1 patients are neuroendocrine gastroenteropancreatic tumors, as they are the most frequent, but also thymic and bronchial carcinoids [8,9,10].

Clinical manifestations of the disease are heterogeneous, as no clear genotype–phenotype correlation has been established. Tumor development may vary in terms of tumor types and the timing of development.

The clinical diagnosis of MEN-1 can be made when at least two out of three of the major endocrine tumors are present (parathyroid tumors and primary hyperparathyroidism, pancreaticoduodenal NETs, and pituitary tumors).

The familial form is diagnosed when there is

- At least one case in the family and at least one first-degree relative with a major endocrine tumor; or
- At least two first-degree relatives with germline pathogenic variant mutation [11].

The identification and early detection of MEN1-related tumors are crucial for timely intervention and improved patient outcomes.

### 2.1. Parathyroid Adenomas

More than 95% of MEN-1 patients develop primary hyperparathyroidism (PHPT), with asymptomatic hypercalcemia being the most common manifestation and earliest presenting symptom [12,13]. MEN-1-associated primary hyperparathyroidism is typically caused by asynchronous and asymmetrical gland hyperplasia or by the development of non-cancerous tumors of the parathyroids, known as parathyroid adenomas [14]. Typically, the disease affects multiple glands to an extent that may vary widely between glands. Adenomas cause the glands to become overactive and to produce excessive amounts of PTH, resulting in increased levels of calcium in the blood (hypercalcemia), which can lead to various symptoms and complications, including bone and abdominal pain, nephrolithiasis or nephrocalcinosis (25% of the patients), digestive issues, and neuropsychiatric symptoms.

By the age of 50, 80–100% of the patients develop parathyroid tumors.

#### 2.1.1. Screening

Screening for primary hyperparathyroidism includes annual plasma calcium and PTH testing starting at age eight (Table 1) [5,15].

Primary hyperparathyroidism presents in two forms:

- The most common presentation is with elevated serum calcium because of PTH hyperproduction that leads to the reabsorption of ionic calcium in kidneys and its release into the bloodstream from increased osteoclast activity within the bone matrix. In patients with suspected hypercalcemia, the American Association of Endocrine Surgeons guidelines recommend dosing with serum calcium, PTH, creatinine, and 25-hydroxyvitamin-D levels [16];
- Normocalcemic presentation is characterized by normal serum calcium values despite elevated PTH values. This condition is also known as incipient PHPT since it may be only a prelude to hypercalcemic PHPT, as progression has been observed in up to 20% of patients [17].

It is essential to consider other differential diagnoses of increased PTH, such as kidney disease (where elevated serum creatinine levels are found), vitamin D (25-hydroxyvitamin D) deficiency, and familial hypocalciuric hypercalcemia (where the 24-hour urinary calcium excretion is normal or low) [16].

#### 2.1.2. Treatment

The treatment is a conventional open bilateral exploration with subtotal parathyroidectomy (more than 3.5 glands need to be removed, otherwise the risk of persistent or recurrent disease is high) or total parathyroidectomy [18,19].

#### 2.1.3. Imaging Features

Preoperative imaging has a limited role in the localization of parathyroid adenomas, which may sometimes be ectopic, making it necessary to examine all four glands surgically despite imaging findings; however, abnormal ectopic glands are occasionally identified (Table 2). In addition, imaging plays an essential role in patients with persistent hypercalcemia following surgery.

##### Ultrasound

Ultrasound localization of the parathyroid glands is particularly useful for patients experiencing persistent or recurrent hyperparathyroidism, as repeat surgery has a high failure rate [18,20].

At ultrasonography, parathyroid adenomas typically appear as well-defined, ovoid, homogeneously hypoechoic nodules compared to the overlying thyroid tissue, located posterior to the thyroid gland. In the case of larger glands, they may show multiple lobes or echogenic regions [15].

The sensitivity of ultrasound in the detection largely depends on the sonographer’s experience and ranges from 72% to 89% [21]. The sensitivity of detecting an adenoma in patients who have undergone previous parathyroidectomy is lower (57%) [22].

Specificity is influenced by the sonographic characteristics of the adenoma. The presence of one or more feeding vessels visualized using color Doppler imaging improves ultrasound specificity for detection from 40% to 93% [23].

**Figure 2:**
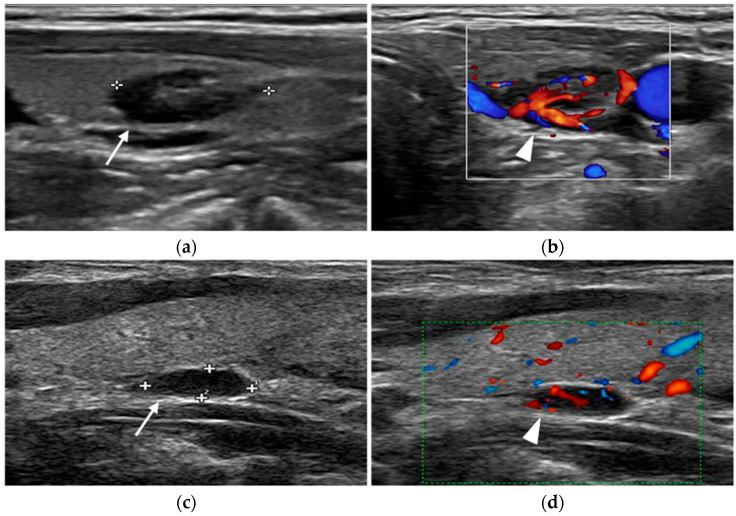
Parathyroid adenomas. In two different patients, ultrasound shows a markedly hypoechoic nodule (arrow) close to the left lobe (**a**) and right (**c**) thyroid lobe. Color-doppler images highlight the feeding vessels (arrowhead) inside the lesions (**b**,**d**).

During an ultrasound examination, the patients lie supine with a pillow under their shoulders to extend the neck. Gray-scale imaging is performed using a high-frequency linear transducer. The examination encompasses the transverse plane from the carotid arteries to the midline and the craniocaudal plane from the hyoid bone or carotid bifurcation to the thoracic inlet. Parathyroid images are both obtained in transverse and longitudinal views. Color Doppler and power Doppler imaging techniques are used alongside gray-scale ultrasound to enhance the detection of prominent feeding arteries (Figure 2).

##### Nuclear Medicine

Functional imaging techniques for studying the parathyroids include planar scintigraphy or single photon emission CT with 99mTc-sestamibi of the neck and mediastinum. The mechanism behind these techniques is the higher uptake and retention of the radiotracer by the parathyroid tissue compared with the surrounding thyroid tissue.

**Figure 3:**
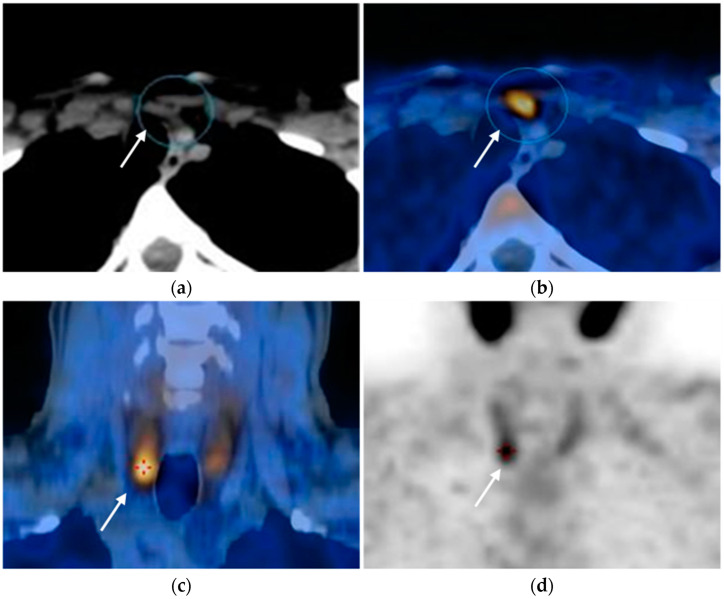
Parathyroid adenomas. 18F-fluorocholine PET/CT (**a**,**b**) and parathyroid scintigraphy (**c**,**d**) of two different patients show radiotracer uptake within parathyroid glands (arrows).

The examination is performed in two phases: in the early phase (15 min after radiotracer injection), the radiotracer accumulates in both thyroid and parathyroid tissue, with higher avidity for the adenomatous parathyroid tissue. In the late phase (about 2–3 h after injection), the hyperfunctioning parathyroid tissue retains the radiotracer (Figure 3).

SPECT/CT further enhances sensitivity in detecting hyperfunctioning parathyroid tissue.

The sensitivity of scintigraphy alone ranges from 77% to 85%, and it is markedly improved (98%) if performed together with a second imaging modality, such as MRI or US [22,24].

Another functional imaging modality that can be performed is 18F-fluorocholine PET/CT, which allows lower radiation exposure, higher resolution, and shorter acquisition times, with a sensitivity of 95%, a PPV of 97%, and a detection rate of 91% [15,25].

Functional imaging is particularly useful as a preoperative modality for accurately localizing parathyroid adenomas, especially if they are ectopic and not identified by ultrasound, either at the time of the first surgery or in case of re-intervention due to the recurrence or persistence of hyperparathyroidism. Preoperative imaging improves surgical success rates from 62% to 97%, thereby reducing morbidity and mortality [20,22].

##### CT

CT is not a first-line examination to assess the parathyroids but may be indicated when secondary imaging is required, especially for surgical planning.

CT can help detect ectopic glands in locations not visible on ultrasound, such as the mediastinum and behind the trachea in the neck [26]. The use of contrast-enhanced, thin-slice CT (2–3 mm) improves the detection of mediastinal ectopic glands.

CT imaging is performed with a field of view (FOV) extending from the maxilla to the carina, usually with a three-phase protocol (unenhanced, arterial, and venous/delayed), as the enhancement patterns of adenomas are variable. In the unenhanced stages, adenomas are always hypoattenuating compared to thyroid tissue. Three different enhancement patterns are recognized:

- Type A enhances more avidly than the thyroid in the arterial phase;
- Type B remains hypodense in both arterial and delayed phases;
- Type C shows less avid enhancement than the thyroid on both arterial and delayed phase images, with greater attenuation than the thyroid in the delayed phase [27].

This method is used in cases of persistent hyperparathyroidism or post-surgery recurrence, for which the sensitivity of detection is 77% [17,28].

##### MRI

Like CT, MRI is not a first-line imaging modality for identifying parathyroid adenomas due to the high sensitivity and specificity of more affordable alternatives like ultrasound and functional imaging. However, MRI is performed in cases of persistent or recurrent hypercalcemia after parathyroidectomy to precisely locate residual hyperfunctioning parathyroid tissue.

**Figure 4:**
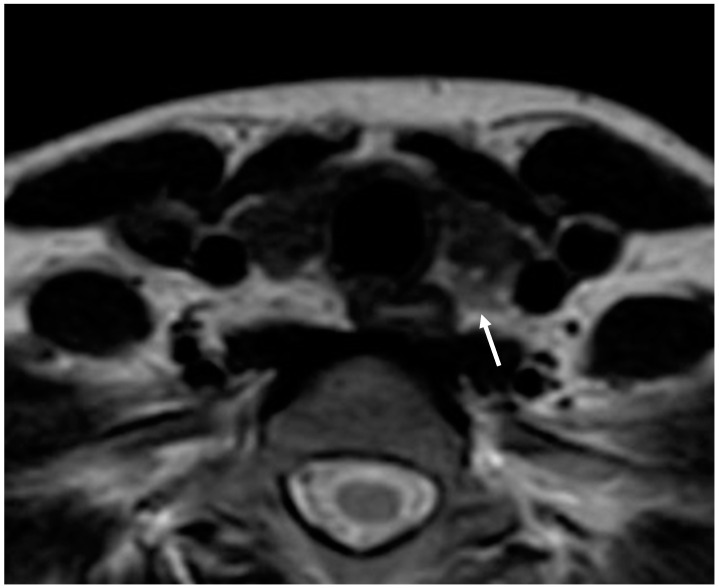
Parathyroid adenoma. Axial T2-weighted image shows a small iso-hyperintense mass behind the left thyroid lobe (arrow).

Parathyroid adenoma is usually seen as a mass with a uniform intermediate-to-high signal on T2-weighted sequences and low signal intensity on T1-weighted sequences. The routine use of MRI contrast agents is not firmly established (Figure 4).

MRI has a variable accuracy for detecting hyperfunctioning parathyroid tissue, with reported sensitivities ranging from 39 and 94%, and specificity of 82–85% [29]. The combination of MRI and 99mTc-sestamibi scintigraphy increases sensitivity to 94% [24]. Furthermore, MRI offers advantages over CT, such as there being no ionizing radiation or the need for iodinated contrast media.

##### Selective Venous Sampling

Venous sampling is a minimally invasive procedure that compares serum PTH levels from various venous territories with those from peripheral blood, achieving a sensitivity of 94%. It can be performed to identify adenomatous tissue when noninvasive imaging methods fail to localize the source of hyperparathyroidism [30].

### 2.2. Pancreatic Neuroendocrine Tumors (pNETs)

In the context of MEN-1 syndrome, pancreatic neuroendocrine tumors (pNETs) are the most common neoplasms after parathyroid adenomas, occurring in 30–80% of patients, and represent a leading cause of MEN-1-related death. Due to their high prevalence and malignant potential in MEN-1 patients, screening for pNETs is essential to facilitate early diagnosis and intervention, ultimately reducing morbidity and mortality.

Depending on their hormone production, pNETs can be classified as non-functioning and functioning.

The majority (90%) of MEN-1-related pNETs are non-functioning and do not cause distinct clinical syndromes [31]. The reason for their non-functionality may lie in the fact that they do not produce hormones at all, produce hormones but do not secrete them, or produce clinically inert pancreatic polypeptides in such small quantities that they do not cause symptoms.

Generally, non-functioning tumors have no clinical symptoms or biochemical changes and are usually detected by imaging examinations [6].

In contrast, functioning pNETs are associated with functional symptoms related to the secretion of functioning hormones. They include insulinomas (the most common), gastrinomas, glucagonomas, VIPomas, somatostatinomas, and PPomas.

When associated with MEN-1 syndrome, they are often multifocal, involving the pancreas and duodenum, and they show higher morbidity and mortality [6,17,32].

Gastrinomas are most frequently malignant, and half of patients have metastases at diagnosis. The clinical manifestation of gastrinoma is Zollinger–Ellison syndrome, characterized by hypergastrinemia leading to multiple and recurrent peptic ulcers and diarrhea.

Insulinomas are typically benign and are characterized by Whipple’s triad: hypoglycemia, neuroglycopenic symptoms (including seizures), and symptom resolution following glucose administration.

#### 2.2.1. Screening

Screening for pancreaticoduodenal NETs includes annual biochemical tests for the following (Table 1):

- Insulin and fasting blood glucose levels from the age of 5;
- Glucagon, VIP, PP, and chromogranin A from the age of 10;
- Gastrin from the age of 20 [11].

Currently, there is no established consensus on the optimal imaging screening program, as it depends on local resources, clinical expertise, and patient preferences. To reduce the morbidity and mortality associated with these tumors, the suggested minimum protocol imaging includes at least annual pancreaticoduodenal imaging with MRI, CT, or EUS, from, at the latest, 16 years old or as soon as clinical symptoms appear (Table 1) [11,33].

#### 2.2.2. Treatment

The treatment for a functioning NET depends on its histotype. Insulinomas are always going to be resected because of the symptoms. For gastrinomas, the therapeutic approach is controversial since patients affected show excellent long-term survival even without surgery. Surgery is usually indicated in the case of less common tumors (VIPoma, Somatostatinoma, Glucagonoma). Non-functioning NETs smaller than 2 cm typically show indolent behavior, so a “watch and wait” approach is adopted, which consists of long-term follow-up to monitor their growth and the possible appearance of symptoms, whereas tumors larger or equal to 2 cm are usually resected [11]. Radiofrequency ablation may also be an option for small NETs [34].

#### 2.2.3. Imaging Features

##### Ultrasound

Abdominal ultrasound is often the first-line imaging modality due to its wide availability and lack of ionizing radiation. However, the sensitivity for the detection of pNET is low (50–60%), especially if tumors are smaller than 2 cm [17]. Sensitivity also depends on the physician’s experience and technical-anatomical conditions related to the patient, such as abundant bowel gas that can make it challenging to evaluate the pancreas or increased liver steatosis that might mask hyperechoic liver metastases [35].

On ultrasound, the tumor usually appears as a solitary, round, well-circumscribed solid mass with smooth margins, hypoechoic compared to normal pancreatic parenchyma.

Larger lesions may show increased heterogeneity due to calcifications or cystic degeneration. During contrast-enhanced ultrasound (CEUS), the tumors appear hypervascular, showing a rapid and intense enhancement in the early phases of the exam; the enhancement is heterogeneous when lesions contain necrotic or hemorrhagic components [36].

Endoscopic ultrasound shows higher sensitivity (93%) than abdominal US, representing the best modality for detecting small pNETs.

Although the sensitivity of CT has increased over time, many small lesions are still missed. Kashab et al. showed that EUS could detect 91% of CT-negative PNETs, and combining the two imaging techniques would probably detect all pNETs [37].

EUS provides information about the size and site of the tumor, and the presence of loco-regional metastases. Moreover, the possibility of performing an EUS-guided biopsy allows the histological characterization of the lesion through needle sampling [17].

EUS is an expensive and time-consuming technique, and the sensitivity depends on the physician’s experience. Moreover, the sensitivity decreases from the head to the tail of the pancreas [33].

At EUS, the tumor usually appears as a solid, heterogeneous, hypervascular mass with hyperenhancement after contrast administration.

Liver metastases are usually hyperechoic compared to normal liver parenchyma; in some cases, they are hypoechoic or show a targetoid appearance [38].

##### CT

Contrast-enhanced CT is one of the most frequently used imaging techniques for localization identification, staging, and follow-up of pNETs. It is a fast and widely available examination but exposes patients to high levels of ionizing radiation.

On basal scans, pNETs are usually isodense to the pancreatic parenchyma.

After the intravenous administration of contrast agents, the hypervascularization of pNETs is reflected by the typical hyperenhancement in the arterial phase (25–30 s after contrast administration). In some cases, the lesions appear hyperdense in the portal venous phase (60–70 s after contrast administration) [32].

In the presence of hemorrhagic and cystic components, the enhancement of the lesions will be heterogeneous [17].

The metastases of pNETs, typically to the liver, usually show the same hyperenhancement in the arterial phase as the primary tumor.

Manta et al. considered a large series of pancreatic neuroendocrine tumors that underwent both CT and EUS with fine needle aspiration, showing a higher sensitivity for EUS compared to CT. In their analysis, CT showed a sensitivity of nearly 64% for recognizing pNETS, failing to detect the lesion in more than 68% of p-NETS smaller than 1 cm and in 15% of patients with a p-NET with a diameter ranging between 1 and 2 cm. CT also overlooked the pancreatic lesion in 46% of cases. In conclusion, the study strongly suggests performing EUS when the clinical suspicion is high, even if the CT is negative [39].

##### MRI

Contrast-enhanced MRI allows the detection of pNETs with higher sensitivity (65–85%) compared to contrast-enhanced CT.

**Figure 5:**
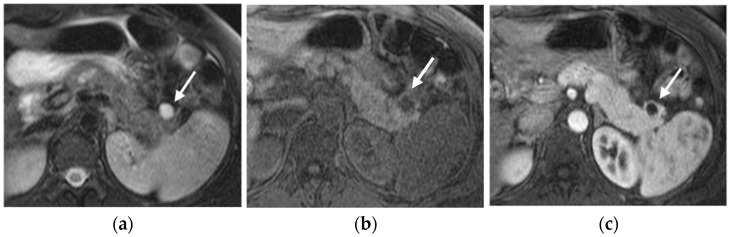

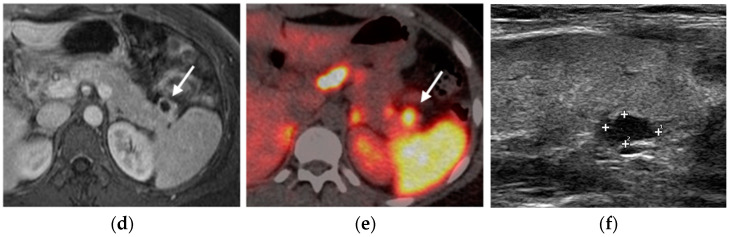
Cystic pancreatic NET of the tail (arrows). Fat-sat T2-weighted image (**a**) and T1-weighted image (**b**) show a cystic-like lesion in the pancreatic tail with a thick and irregular enhancing wall (**c**,**d**). PET-CT highlights high tracer uptake of the lesion (**e**). In the same patient, ultrasound revealed a synchronous parathyroid adenoma (**f**).

The specific MRI appearance of PNETs can be influenced by factors such as tumor grade, size, and the presence of cystic or necrotic areas within the lesion [40] (Figure 5).

Compared to the surrounding pancreatic tissue, pNETs usually appear isointense/hypointense on T1-weighted MRI sequences and variably hyperintense on T2-weighted MRI sequences, depending on the amount of collagen in the tumor [32].

**Figure 6:**
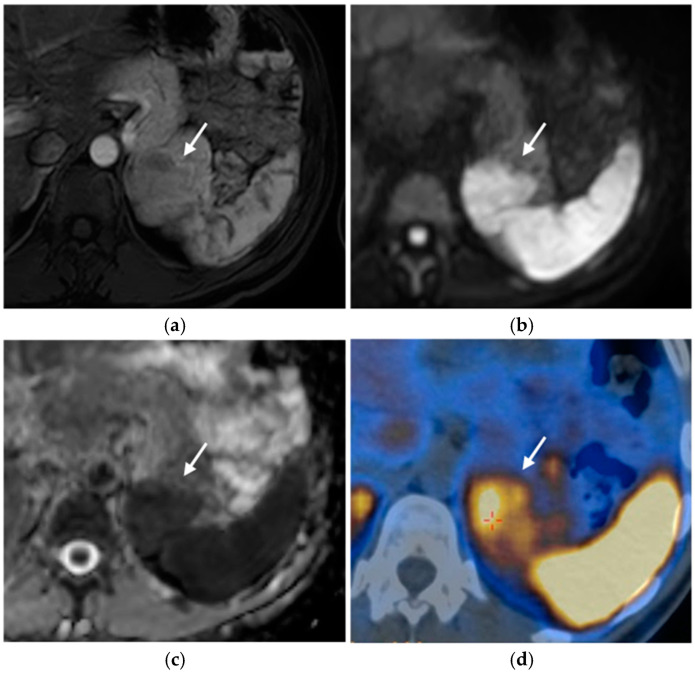
Insulinoma. Heterogeneously enhancing mass (arrows) in the pancreatic tail (**a**), with significant diffusion restriction (**b**,**c**) and avid uptake on PET-CT (**d**).

PNETs exhibit varying signal intensity with Diffusion-Weighted Imaging (DWI), but they often demonstrate restricted diffusion, with smaller lesions potentially being not easily detected on other MRI sequences [17]. Less differentiated tumors demonstrate even more diffusion restriction (Figure 6).

After contrast administration, pNETs show hyperintensity in the arterial phase.

Advanced pNETs may demonstrate local invasion into surrounding structures, such as blood vessels, adjacent organs, or the pancreatic duct. MRI can help detect these features and evaluate the extent of local invasion.

Additionally, MRI is useful for detecting liver metastases, which are common in pNETs, and defining their size and distribution. They usually appear hyperintense in T2-weighted images and show enhancement in the early arterial phase.

Some uncommon patterns include pNETs showing a mixed appearance with areas of varying signal intensity on a T2-weighted MRI, reduced enhancement in all contrast phases, or peripheral enhancement with a gradual filling of the lesion [41].

##### Nuclear Medicine

Functional imaging exams are whole-body nuclear medicine techniques employed to stage tumors and characterize the primary tumor in cases where pNET is detected as metastatic disease.

Functional imaging has no role in screening. The ENETS guidelines recommend performing Ga-PET-DOTATOC PET-CT for staging and assessing somatostatin receptors when there is a known pNET of at least 1 cm or that shows growth [33,42,43].

**Figure 7:**
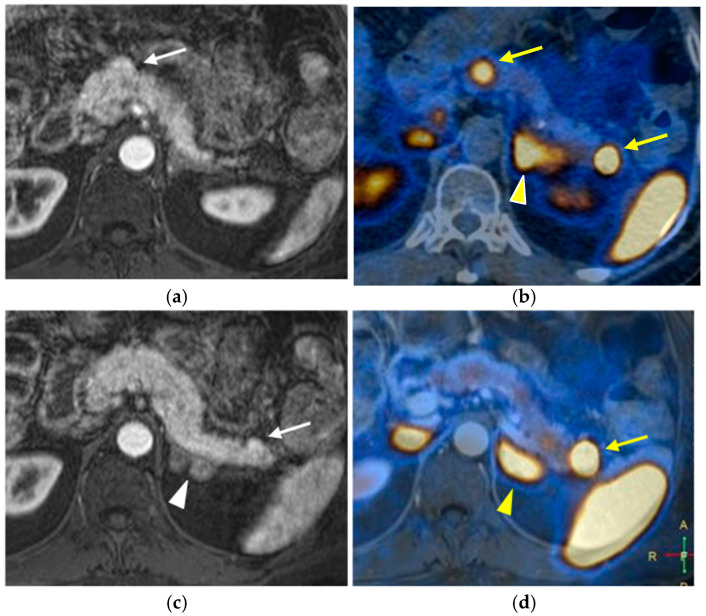
Synchronous non-functioning pancreatic neuroendocrine tumors and adrenal adenomas. Arterial phase contrast-enhanced T1-weighted images show two slightly hyperenhancing nodules (white arrows) in the pancreatic isthmus (**a**) and in the tail (**c**). Nodular thickening of the left adrenal gland (white arrowhead) was also found (**c**). Subsequent 68Ga-DOTATOC-PET-CT (**b**,**d**) demonstrated high uptake by the pancreatic (yellow arrows) and adrenal lesions (yellow arrowhead).

Somatostatin-receptor-scintigraphy has been gradually replaced by PET-CT, which offers higher diagnostic accuracy, lower radiation dose, decreased costs, and a shorter examination time [44]. These imaging methods utilize radiotracers, such as positron-emitting 68Ga–tetraazacyclododecanetetraacetic acid (DOTA). 68Ga-DOTATOC, 68Ga-DOTATATE, and 68Ga-DOTANOC specifically bind to somatostatin receptors, which are found in significantly higher concentrations on the surface of neuroendocrine tumor cells compared to surrounding tissues [17,32] (Figure 7).

FDG is another common radiotracer that accumulates in glucose-consuming tissues; therefore, in PET-FDG studies, a higher signal is related to highly proliferating and aggressive tumors. The tracer enables the detection of less differentiated neuroendocrine tumors that do not uptake other radiotracers, but it is not very specific. Another disadvantage of using FDG is that it misses carcinoid tumors and small neoplasms (<1 cm) [45].

##### Angiography

Angiography may be considered in selected cases when clinical symptoms or laboratory abnormalities are present, but the tumor cannot be localized with non-invasive imaging methods. The Doppman test [46], also known as the selective arterial secretagogue injection test, involves the injection of secretagogues (e.g., calcium gluconate) into specific arteries supplying the pancreas while monitoring hormone levels in the hepatic venous blood. The secretagogue stimulates hormone release from the tumor only when injected into its feeding vessel, helping to pinpoint the tumor’s location. The Doppman test is especially useful for identifying insulinomas and gastrinomas. By providing a precise localization, the test enables surgical planning and helps to identify the hormonally active lesion in patients with multiple pNETs [47].

### 2.3. Pituitary Adenomas

Pituitary adenomas occur in 15–50% of patients with MEN-1 syndrome, appearing more frequently in women than men [6].

Pituitary adenomas arising in patients with MEN-1 syndrome are usually more aggressive than the sporadic ones, have larger dimensions, increased invasiveness, and poor response to treatment, and thus require a close biochemical follow-up [48].

According to their size, it is possible to distinguish between microadenomas (60%), with a maximum diameter within 10 mm, and macroadenomas (40%), larger lesions that frequently show extrasellar extension and that are present with visual impairment or hypopituitarism.

Pituitary adenomas can also be divided into functioning or non-functioning, according to the secretion of different hormones. The most frequent are prolactin-secreting adenomas (60%), which usually affect young women presenting with galactorrhea and secondary amenorrhea. Affected men may show erectile dysfunction and decreased libido, while children typically manifest delayed puberty and primary amenorrhea. Other functioning adenomas include somatotrophinomas (24%, secreting growth hormone alone or growth hormone and prolactin, resulting in acromegaly) and adrenocorticotrophinomas (<5%, secreting ACTH, resulting in Cushing disease) [5,49]. Less than 15% of pituitary adenomas are non-functioning, most commonly microadenomas.

#### 2.3.1. Screening

Clinical Practice Guidelines (2012) suggest starting screening at age five (Table 1). The first pituitary MRI is recommended between ages 5 and 10. For patients without pituitary adenoma, a biochemical screening (annual assessment of plasma prolactin and IGF-I levels) and pituitary MRI every 3 years are recommended [11].

#### 2.3.2. Treatment

The treatment of pituitary adenoma is the same as for subjects not affected by MEN-1 syndrome. It may include medical (e.g., dopamine agonists for prolactinomas) or surgical (hypophysectomy with a transsphenoidal approach) therapy. Radiotherapy can also be used in case of residual unresectable tumor tissue [5].

#### 2.3.3. Imaging Features

##### CT

The role of CT in the initial evaluation of pituitary adenomas is limited due to the radiation exposure and the isodensity of the lesions compared to the surrounding brain tissue. However, CT may be performed when MRI is contraindicated: it is useful in detecting soft tissue calcification, bony destruction (as larger tumors may cause bone remodeling of the sella turcica), and investigating bony anatomy before surgery. The enhancement of adenomas is variable, but most of them show marked enhancement [50].

##### MRI

MRI is the gold standard imaging modality for diagnosing and following pituitary adenomas: its excellent soft tissue contrast helps characterize pituitary lesions and their relationship with surrounding structures, providing useful information for surgical planning.

The MRI protocol for investigating hypophyseal diseases includes small-field-of-view coronal and sagittal T1-weighted and T2-weighted sequences, focused on the sellar region, as well as coronal and sagittal T1-weighted sequences after intravenous contrast administration [49]. Dynamic post-contrast T1-weighted sequences may be helpful in the detection of microadenomas.

**Figure 8:**
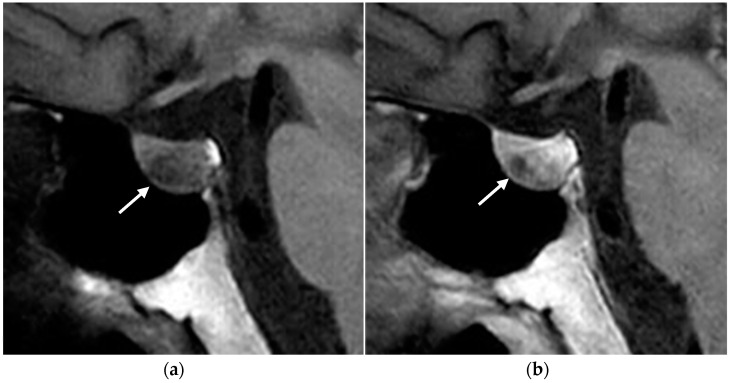
Pituitary micro-prolactinoma. T1-weighted MR images before (**a**) and after (**b**) contrast administration showing a hypointense and low-enhancing nodule (arrow) in the anterior pituitary gland.

Adenomas are often isointense to the adjacent pituitary parenchyma in T1- and T2-weighted sequences. However, microadenomas can be slightly hyperintense on T2-weighted images and have a delayed enhancement compared to the background pituitary parenchyma (Figure 8). Some small microadenomas are not always visualized on imaging, and therefore it is important to acquire dynamic post-contrast sequences, as they increase the sensitivity in identifying the lesion, and to look for secondary signs, such as erosion of the sellar floor or focal convexity of the superior pituitary gland [17].

**Figure 9:**
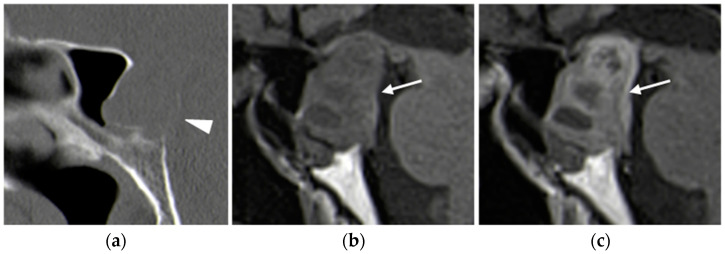
Pituitary macro-adenoma. Sagittal plane basal CT (**a**) image shows a large mass causing bone remodeling of the sella turcica and thinning on the posterior wall of the sphenoid sinus (arrowhead). MRI T1-weighted images before (**b**) and after (**c**) contrast administration confirm the presence of a large hypointense lesion (arrows) arising from the pituitary gland, with heterogeneous enhancement.

Pituitary macroadenomas can have a heterogeneous signal on unenhanced sequences due to solid, cystic, necrotic, and hemorrhagic areas. After contrast administration, macroadenomas typically show heterogeneous enhancement (Figure 9).

MRI intensity patterns have been studied to better characterize different subtypes of pituitary adenomas: a study from Gruppetta reports that hypointensity on T2-weighted sequences is more frequent in GH-secreting pituitary adenomas, compared to other adenoma subtypes; in addition, among GH secreting pituitary adenomas, hypointense T2-weighted ones showed higher IGF-1 levels, a smaller tumor size, less tumor invasiveness, and a better response to somatostatin analogue treatment [51].

### 2.4. Thymic and Bronchial Neuroendocrine Tumors (Carcinoids)

In the context of MEN-1 syndrome, other rare tumors can be found, such as thymic and bronchial carcinoids.

Thymic carcinoid is usually aggressive and is typically detected at an advanced stage because of local symptoms or by chance on imaging, as it more frequently remains asymptomatic. It affects 8% of MEN-1 patients and has a male prevalence. Half of the tumors are functional, secreting ACTH and causing Cushing’s syndrome. It is recommended to screen these patients by performing a CT or MRI scan of the chest every 1 or 2 years to identify the disease as early as possible (Table 1).

Altemir Trallero et al. suggested performing an annual CT or MRI to achieve an early diagnosis of thymic carcinoid and to perform total thymectomy during the same surgical procedure as parathyroidectomy [52].

Bronchial carcinoids affect 5–35% of MEN-1 patients, more commonly males and smokers. Tumors are typically multifocal and it is recommended to perform CT or MRI of the chest every 1–2 years [5,11] (Table 1).

## 3. Conclusions

MEN-1 is a rare syndrome linked to an increased risk for developing specific hormone-secreting tumors. Effective diagnosis and management of MEN-1 require the collaboration between multiple specialists for genetic testing, regular surveillance, and imaging diagnosis and follow-up. Radiologists should be aware of the most frequent MEN-1-associated neoplasms and their imaging features to provide the best information for an accurate diagnosis, management, and treatment.

## Figures and Tables

**Table 1:** Screening modalities in MEN-1.

	Screening Modality	Interval	Starting Age
Parathyroid adenoma	- Plasma calcium and PTH	Yearly	8 years
Pancreatic neuroendocrine tumors	- Insulin and fasting blood glucose levels	Yearly	5 years
	- Glucagon, VIP, PP, and chromogranin	Yearly	10 years
	- Gastrin	Yearly	20 years
Pituitary adenoma	- Pituitary MRI	3 years	5 years
	- Prolactin and IGF-I levels	3 years	5 years
- Thymic carcinoid	Chest CT/MRI	1–2 years	Not defined
- Bronchial carcinoid	Chest CT/MRI	1–2 years	Not defined

**Table 2:** Imaging modalities for the most common tumors in MEN-1.

	Ultrasound	CT	MRI	Nuclear Medicine	Angiography
Parathyroid adenoma	72–89% Sens. Hypoechoic.	Ectopic parathyroid detection. Hypoattenuating on native. Variable enhancement.	Sens 39–94%, Spec 82–85%. High T2 signal.	99mTc-sestamibi. 77–85% Sens.	Sens 94%. Venous sampling.
Pancreatic neuroendocrine tumors	First-line; 50–60% Sens. Hypoechoic.	64% Sens. Hypervascular in arterial phase.	65–85% Sens. Hyper on T2; hypervascular in arterial phase.	Radiotracer connecting to somatostatine receptors. Sens higher than CT.	Hormone levels sampling. Last chance.
Pituitary adenoma	No role	Only if MRI is absolutely contraindicated.	Almost isointense on T1/T2. DCE post-contrast	No role	No role

## Data Availability

Not applicable.
